# The Importance of Clinical Reasoning in Pancreatic Insufficiency: A Case Report

**DOI:** 10.7759/cureus.38760

**Published:** 2023-05-09

**Authors:** Jonathan Otero-Colón, Yisia Olivero, Parmbir Virk, Madilyn Thomas, Samantha Webking, Jose Mejia

**Affiliations:** 1 Internal Medicine, Nassau University Medical Center, East Meadow, USA

**Keywords:** acute-on-chronic pancreatitis, alcoholic pancreatitis, pancreatic insufficiency, exocrine, epi

## Abstract

Evidence-based medicine has demonstrated an extensive list of etiologies for exocrine pancreatic insufficiency (EPI). EPI is defined as inadequate pancreatic enzyme efficacy in digestion due to insufficient enzyme production, activation, or early enzyme degradation. Among the etiologies, acute pancreatitis secondary to chronic and excessive consumption of alcohol has been found to be one of the most common causes. In 2022, a 43-year-old male patient with a past medical history of polysubstance abuse, acute on chronic pancreatitis, alcohol dependence, pulmonary embolism, hypertension, hyperlipidemia and diabetes mellitus type 2 presented to the Emergency Department with three days of epigastric abdominal pain, nausea and non-bloody, non-bilious vomiting. Proper imaging confirmed the diagnosis of acute pancreatitis. The key to treatment and surveillance relies on proper identification of risk factors, pertinent imaging for diagnostic evaluation and appropriate treatment with electrolyte repletion. The patient developed persistent electrolyte deficiencies despite appropriate repletion, indicating high suspicion of pancreatic insufficiency. The treatment most importantly relies on a combination of repletion of electrolytes as well as pancreatic enzymes with a clear patient understanding of their chronic condition, the importance of reducing modifiable risk factors and compliance with medical therapy.

## Introduction

Exocrine pancreatic insufficiency (EPI) is defined as inadequate pancreatic enzyme efficacy in digestion due to insufficient enzyme production, activation, or early enzyme degradation [1]. EPI development is associated with a multitude of pathological conditions including autoimmune pancreatitis, celiac disease, inflammatory bowel disease, Diabetes mellitus, pancreatic tumors, cystic fibrosis, and alcoholic-associated chronic pancreatitis [1,2]. The probability of exocrine insufficiency increases with disease duration and it occurs more rapidly in alcohol-related pancreatitis. The majority of patients with alcohol-associated chronic pancreatitis present EPI within 10 years after diagnosis and almost all after 20 years [2]. EPI develops in the setting of chronic pancreatitis when the destruction of the pancreatic parenchyma reaches around 90% [1]. The common clinical presentation of chronic pancreatitis is diffuse upper abdominal pain that may radiate to the back. There is an associated increase in pain with meals that tends to decrease both appetite and food consumption, often resulting in weight loss [3]. In the case of EPI secondary to chronic pancreatitis, clinical findings may also include steatorrhea, multiple vitamin and micronutrient deficiencies, and electrolyte abnormalities [4,5].

Various direct and indirect pancreatic function tests are available to diagnose EPI. Direct testing includes the most sensitive and specific methods such as secretin-magnetic pancreatography and secretin-endoscopic ultrasonography; however, these tests are very expensive and often only performed at specialized centers [1]. Indirect testing is non-invasive and assesses the effects of pancreatic enzymes in the gastrointestinal tract such as undigested food or the presence of enzymes. Treatment of EPI varies on etiology, but in the case of alcohol-related pancreatitis, complete abstinence from alcohol is essential to minimize the progression of the disease along with vitamin supplementation and pancreatic enzyme replacement [6]. The objective is to deliver sufficient enzymatic activity into the duodenal lumen simultaneously with the meal in order to optimize digestion and absorption of nutrients [1]. Our case presents a patient with undiagnosed EPI causing resistant electrolyte deficiencies.

## Case presentation

**Figure 1:**
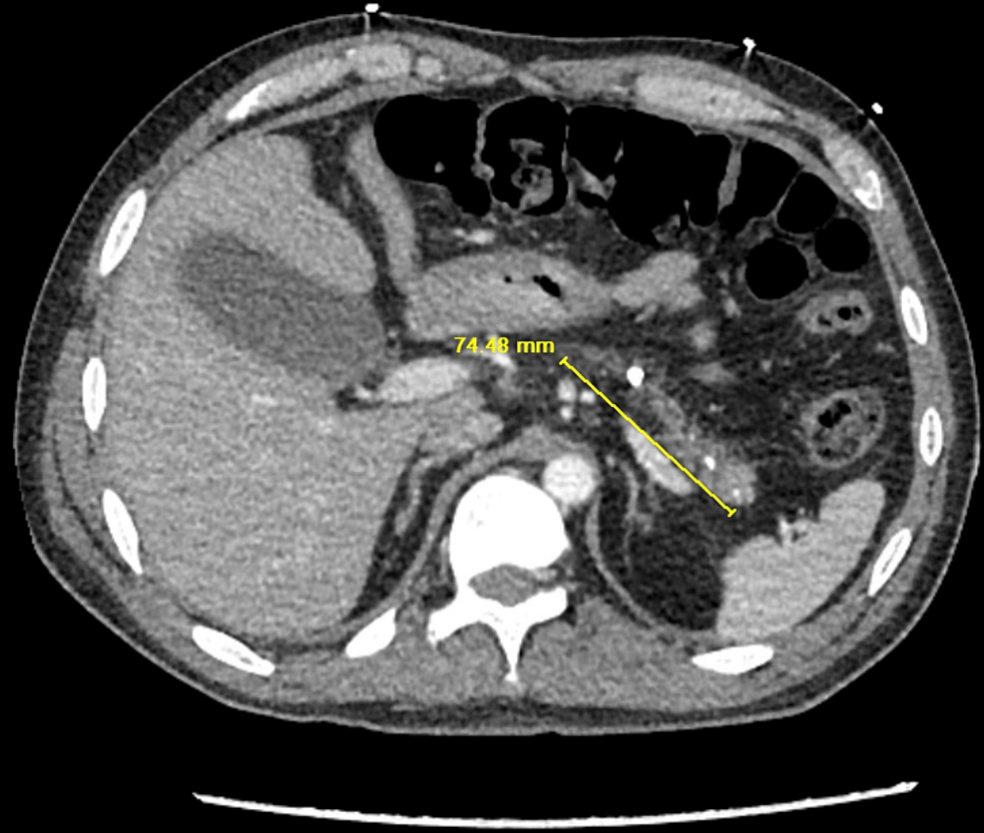
Diffusely atrophic pancreas with numerous parenchymal calcifications.

The patient is a 43-year-old male with a past medical history of polysubstance abuse, alcohol dependence, acute pancreatitis, pulmonary embolism, hypertension, hyperlipidemia and diabetes mellitus type 2 presenting to the Emergency Department with three days of epigastric abdominal pain, nausea and non-bloody, non-bilious vomiting. Past history is significant of multiple admissions for chronic alcohol dependence and various episodes of diagnosed pancreatitis. CT scan of the abdomen and pelvis with contrast was performed and demonstrated a severely atrophic pancreas with numerous parenchymal calcifications which is compatible with sequelae of chronic pancreatitis (Figure 1). The need for electrolyte and nutritional monitoring is critical with the confirmation of chronic pancreatitis.

The patient's evaluation demonstrated hypokalemia, hypomagnesemia, hypocalcemia, and hypophosphatemia. Repletion was performed through oral and intravenous routes. Despite adequate repletion, hypomagnesemia and hypokalemia demonstrated to be resistant to treatment. Symptoms were accompanied by occasional nausea, vomiting and diarrhea. Throughout the patient’s hospital course, a cyclical pattern emerged of increases in electrolyte concentrations shortly after oral and intravenous administration, with consecutive electrolyte derangement noted thereafter (Tables 1, 2). A trial of pancreatic enzymes was initiated. The diagnosis of pancreatic insufficiency secondary to chronic pancreatitis due to chronic alcohol dependence was achieved. Patient was placed on pancreatic enzyme replacement therapy and discharged with continued treatment. Unfortunately, the patient was found to be non-compliant with therapy and continues to struggle with alcohol dependence.x

**Table 1:** Undulating daily magnesium despite adequate repletion. *Magnesium repletion with magnesium sulfate at 4-8g over 24hrs.

Magnesium (mg/dL)	Dates (M/D/YY)
1.5	8/27/2022
1.3	8/28/2022
2.0	8/29/2022
1.8	8/30/2022
1.5	8/31/2022
2.2	9/1/2022
1.5	9/2/2022
1.7	9/3/2022
1.3	9/4/2022
1.2	9/5/2022
2.0	9/6/2022
1.2	9/7/2022
2.3	9/8/2022
1.5	9/9/2022
1.5	9/10/2022
1.1	9/11/2022
1.9	9/12/2022
2.2	9/13/2022
1.9	9/14/2022
1.3	9/15/2022
1.3	9/16/2022
1.4	9/17/2022
1.7	9/18/2022
1.7	9/19/2022
1.0	9/20/2022
0.9	9/21/2022
1.0	9/22/2022
1.5	9/23/2022
1.2	9/24/2022
0.9	9/25/2022
1.2	9/26/2022
0.9	9/27/2022

**Table 2:** Undulating potassium despite adequate repletion. *Potassium repletion with oral potassium chloride at 40mEq three to four times daily and IV 10mEq per hour up to 40mEq daily.

Potassium (mEq/L)	Dates (M/D/YY)
2.0	8/27/2022
2.1	8/28/2022
2.2	8/29/2022
2.6	8/30/2022
2.4	8/31/2022
2.4	9/1/2022
2.3	9/2/2022
2.8	9/3/2022
2.4	9/4/2022
3.7	9/5/2022
2.5	9/6/2022
3.8	9/7/2022
2.4	9/8/2022
2.3	9/9/2022
5.1	9/10/2022
2.4	9/11/2022
3.1	9/12/2022
2.8	9/13/2022
3.1	9/14/2022
3.0	9/15/2022
4.2	9/16/2022
4.1	9/17/2022
3.9	9/18/2022
3.7	9/19/2022
3.7	9/20/2022
3.4	9/21/2022
3.1	9/22/2022
3.5	9/23/2022
3.2	9/24/2022
2.9	9/25/2022
5.5	9/26/2022
2.6	9/27/2022

## Discussion

Of the various possible origins of acute pancreatitis, chronic excessive consumption of alcohol has been found to be the most common cause and accounts for roughly 50% of cases [7]. Many patients with recurrent episodes of acute pancreatitis related to alcohol use will develop chronic pancreatitis with continued alcohol consumption being a key factor in prognosis [3]. In a cohort study made in the United States, alcohol was estimated to account for half the cases of chronic pancreatitis, and the incidence of disease along with alcohol use has only increased from previous years [8]. In this case, the patient has a chronic alcohol use disorder. The condition has caused many sequelae including EPI.

Pancreatic exocrine insufficiency is one of the long-term consequences of chronic pancreatitis [9]. Advanced EPI results in maldigestion of fat and protein leading to multiple vitamin and micronutrient deficiencies. Furthermore, global malabsorption results from diseases associated with either widespread mucosal involvement or a reduced absorptive surface such as in chronic pancreatic insufficiency to a severe degree [4]. States of global malabsorption of this degree may produce diminished concentrations including serum calcium, magnesium, and zinc. In our case of pancreatic insufficiency secondary to long-term alcohol abuse, laboratory findings revealed severe, resistant hypomagnesemia, hypokalemia, hypophosphatemia, and hypocalcemia.

Magnesium has been identified as a promising marker in the identification of pancreatic insufficiency in patients with chronic pancreatitis. In a retrospective analysis investigating the correlation between different nutritional markers and pancreatic exocrine insufficiency, magnesium was found to have the strongest positive indication [10]. Furthermore, a 2013 prospective study performed by Papazachariou et. al revealed a positive correlation between chronic pancreatitis and magnesium deficiencies in 13 patients [5]. A confirming study was produced with a larger cohort. A 2021 study of 112 patients with pancreatic insufficiency also indicated that magnesium deficiency is significantly associated with pancreatic exocrine insufficiency [11]. Despite the emergence of several studies, expert consensus has been that the evidence is not strong enough to recommend routine monitoring of magnesium levels in patients with pancreatic insufficiency [12,13]. Magnesium levels can serve as a marker of nutritional status and repletion of magnesium in patients with pancreatic insufficiency may prevent serious adverse events, such as hospitalization. Magnesium can be repleted orally and via IV, and the exact route will likely be dictated by the overall clinical picture. Given the potentially serious sequelae of magnesium deficiency, it may be reasonable to monitor magnesium levels in patients with pancreatic insufficiency, but more evidence is necessary before it becomes a routine clinical practice. If the patient is obtaining a CMP or magnesium level for other reasons, the medical practitioner should keep the potential impact of pancreatic insufficiency on micronutrients, including magnesium, in mind when interpreting test results. In addition, hypomagnesemia should be included in the differential diagnosis of patients with pancreatic insufficiency who report neurological symptoms, such as tingling, numbness, fatigue or muscle cramps. This study places emphasis on the importance of having high suspicion for EPI with chronic pancreatitis and resistant electrolyte abnormalities.

## Conclusions

It has been proven through evidence-based medicine that various possible origins exist for acute pancreatitis, but that there is a clear correlation with chronic excessive consumption of alcohol, leading it to become the most common related cause. As defined above, EPI is the inadequate pancreatic enzyme efficacy in digestion due to insufficient enzyme production, activation, or early enzyme degradation. A multitude of pathological conditions including autoimmune pancreatitis, celiac disease, inflammatory bowel disease, diabetes mellitus, pancreatic tumors, cystic fibrosis, and alcoholic-associated chronic pancreatitis result in EPI. EPI develops in the setting of chronic pancreatitis when the destruction of the pancreatic parenchyma reaches around 90%.

In the case of our 43-year-old male with a past medical history of polysubstance abuse, alcohol dependence, acute pancreatitis, and multiple admissions for chronic alcohol dependence leading to the sequelae of acute on chronic pancreatitis with various episodes of acute pancreatitis. Along with imaging, the presence of severe electrolyte abnormalities painted the picture of EPI. Correct steps of electrolyte repletion and treatment with pancreatic enzymes were given and the patient was discharged with a poor prognosis due to a history of non-compliance.

As discussed above in multiple cohort case studies, the relationship between EPI and alcohol abuse was exemplified, as nearly half the cases were shown to have a direct correlation. Furthermore, it was found that these patients also developed alcohol-associated severe electrolyte imbalances due to malabsorption as a result of EPI and surface area disease. Retrospective analysis directly showed a positive correlation of EPI with magnesium imbalance. However, it was recommended that routine monitoring was not necessary. Since the is no clear monitoring for EPI the importance of clinical reasoning is paramount. Magnesium should be used as a marker and used to prevent adverse events in patients with EPI. In conclusion, there is a relationship between chronic alcohol use and EPI, with magnesium deficiency as a strong positive marker, which should be repleted adequately to reduce the number of adverse events and hospitalizations.
